# Anti-gp120 Minibody Gene Transfer to Female Genital Epithelial Cells Protects against HIV-1 Virus Challenge *In Vitro*

**DOI:** 10.1371/journal.pone.0026473

**Published:** 2011-10-21

**Authors:** Ussama M. Abdel-Motal, Phuong T. N. Sarkis, Thomas Han, Jeffery Pudney, Deborah J. Anderson, Quan Zhu, Wayne A. Marasco

**Affiliations:** 1 Department of Cancer Immunology and AIDS, Dana-Farber Cancer Institute, Harvard Medical School, Boston, Massachusetts, United States of America; 2 Department of Obstetrics and Gynecology, School of Medicine, Boston University, Boston, Massachusetts, United States of America; University of Nebraska – Lincoln, United States of America

## Abstract

**Background:**

Although cervico-vaginal epithelial cells of the female lower genital tract provide the initial defense system against HIV-1 infection, the protection is sometimes incomplete. Thus, enhancing anti-HIV-1 humoral immunity at the mucosal cell surface by local expression of anti-HIV-1 broadly neutralizing antibodies (BnAb) that block HIV-1 entry would provide an important new intervention that could slow the spread of HIV/AIDS.

**Methods and Findings:**

This study tested the hypothesis that adeno-associated virus (AAV)-BnAb gene transfer to cervico-vaginal epithelial cells will lead to protection against HIV-1. Accordingly, a recombinant AAV vector that encodes human b12 anti-HIV gp120 BnAb as a single-chain variable fragment Fc fusion (scFvFc), or “minibody” was constructed. The secreted b12 minibody was shown to be biologically functional in binding to virus envelope protein, neutralizing HIV-1 and importantly, blocking transfer and infectivity of HIV-1_bal_ in an organotypic human vaginal epithelial cell (VEC) model. Furthermore, cervico-vaginal epithelial stem cells were found to be efficiently transduced by the optimal AAV serotype mediated expression of GFP.

**Conclusion:**

This study provides the foundation for a novel microbicide strategy to protect against sexual transmission of HIV-1 by AAV transfer of broadly neutralizing antibody genes to cervico-vaginal epithelial stem cells that could replenish b12 BnAb secreting cells through multiple menstrual cycles.

## Introduction

The mechanisms of HIV-1 transmission through the vaginal route in women are still poorly understood. Epithelial cells lining the mucosal surfaces of the female genital tract provide the first line of defense against sexually transmitted pathogens such as HIV-1 [1], [2]. The multilayer squamous cell epithelia lining the vagina and ectocervix provide a more substantial barrier against HIV-1 invasion than the single layer columnar epithelium that lines the endocervix [3]. Epithelial cells also produce several biological factors, such as defensin, lactoferrin and secretory leukocyte protease inhibitor (SLPI) that have anti-HIV properties [1], [3], [4], [5], [6]. However, any damage or disruption to the epithelial layer, which can occur as a result of inflammation from sexually transmitted diseases (STDs) or even mild trauma during sexual intercourse may increase the ability of HIV-1 to penetrate the mucosal epithelial barrier. In addition, several cell surface receptors and molecules have been reported to facilitate HIV-1 entry into epithelial cells allowing passage through the mucosal barrier. Syndecans (expressed on the vaginal epithelial cells), for example, were found to be exploited by HIV-1 to cross the mucosal epithelium by transcytosis [7], [8], [9], [10]. It has been reported that the Arg298 in gp120 mediates HIV-1 binding to syndecans, and the human b12 anti-HIV gp120 BnAb can block this interaction [8], [11], [12], [13].

The b12 molecule is one of a growing number of human BnAbs including, 2G12, 2F5, 4E10, Z13e1, VRC01, HJ16, PG9 and PG16 that are capable of potently neutralizing a broad range of primary HIV-1 isolates [12], [14]. B12 was originally isolated as an antibody fragment (Fab), which recognizes a highly conserved epitope on the viral gp120 envelope protein involved in binding to CD4 on host cells [12]. In addition, b12 IgG1 can inhibit transfer of cell-free HIV-1 to the ME-180 human cervical epithelial cell line and block viral attachment to and uptake by epithelial cells [15]. Macaques treated with b12 IgG1 by intravenous or intravaginal (topical) application were shown to be protected against simian human immunodeficiency virus (SHIV) infection by the vaginal route [16]. These studies support the choice of b12 mAb to investigate the hypothesis that genetic transfer of a BnAb to cervico-vaginal cells can confer protection from viral infection at the mucosal surface.

Adeno-associated viral (AAV) vectors are capable of transducing a variety of tissues and cell types [17], [18], [19], [20], [21], [22] with the potential of directing long-term expression from months to years since the vector persists predominantly in episomal form [17], [19], [21], [23]. However, the upper layer of the cervico-vaginal mucosa continuously sheds. In contrast, the basal layer of the mucosa including the epithelial stem cells is maintained as a replenishing source of squamous epithelial cells. Accordingly, targeting the genital epithelial stem cells for transduction by AAV would be ideal for stable and durable gene transfer *in vivo*. Therefore, a major aim of this study was to investigate the possibility of transducing female genital epithelial stem cells.

In the present study, an AAV vector was utilized for transfer of the b12 antibody gene to cervico-vaginal epithelial cells. In particular, a recombinant AAV-6 expressing b12 minibody was produced and the minibodies secreted from transduced cells in an organotypic vaginal epithelial cell (VEC) model demonstrated their ability to inhibit transfer and infectivity of HIV-1 at levels comparable to full-length b12 IgG1 MAb. Transduction of primary genital-epithelial stem cells was also demonstrated. The findings of this study demonstrate that use of the AAV vector to express neutralizing human anti-gp120 minibodies is a promising strategy for developing an effective durable microbicide against HIV-1 infection.

## Materials and Methods

### Growth media

Human cervical and VEC lines and huPGECs used in this study were cultured in keratinocyte serum-free medium provided with supplementary bovine pituitary extract and recombinant human Epidermal Growth Factor (EGF). The medium was further supplemented with 100 units/ml penicillin, 100 µg/ml streptomycin and CaC1_2_ to a final calcium concentration of 0.4 mM. DMEM/F12 medium was supplemented with 10% fetal bovine serum (FBS). All media and supplements were obtained from Invitrogen Corp. (Carlsbad, CA).

### Cell lines and viruses

The human cell lines VK2/E6E7, Ectl/E6E7 and Endl/E6E7, originally constructed in the laboratory of Deborah Anderson [24] and obtained from the American Type Culture Collection (ATCC, Manassas, VA), were cultured in calcium-supplemented (0.4 mM) keratinocyte serum-free medium. TZM-bl cells were acquired from the National Institutes of Health AIDS Research and Reference Reagent Program (NIH-ARRRP, Germantown, MD). TZM-b1 cells are a CXCR4-positive HeLa cell line that expresses the cellular receptor (CD4) and co-receptor (CCR5) for HIV-1. The cell line also contains integrated reporter genes for luciferase and *E. coli* beta-galactosidase, under the control of an HIV long-terminal repeat sequence (*tat* gene) which allows for quantification of HIV infection. 293T cells and COS-1 (both from ATCC) and TZM-bl cells were cultured in DMEM medium supplemented with 10% FBS and 1% penicillin/streptomycin (Invitrogen). All cells and cultures were maintained at 37°C in a 5% CO_2_ humidified incubator. The R-tropic HIV-1_bal_ strain (NIH-ARRRP) used for the transwell studies were only passaged in human PBMCs and titered by the standard Reed and Muench method [25].

### Organotypic VEC culture and HIV-1 transmission model

Organotypic EpiVaginal™ tissues (VEC-100: Nunc™ single well tissue culture plate inserts; pore size  = 0.4 µm; inner diameter  = 0.80 cm) were purchased from MatTek Corp. (Ashland, MA) and maintained with proprietary growth medium according to the company's instructions. Fully differentiated stratified squamous epithelial layers were maintained on the cell culture insert membranes to allow growth at the air–liquid interface. The integrity of the VEC tissues on the transwell filters were verified using Trans Epithelial Electric Resistance (TEER) readings (>300 ohm) and permeability to 70 kDa Dextran-Rhodamine B (Sigma Corp., St. Louis, MO) applied to the apical layer of the tissue and measuring paracellular passage of the dye into the lower transwell chamber. We adapted the procedure by Bobardt *et al*., 2007 to examine the effects of the b12 minibodies or of AAV-6 expressing b12 minibody transduction on HIV-1 transfer through the multilayered VEC tissue. The b12 minibodies or full-length b12 IgG1 (10 µg/ml) were pre-incubated with or without HIV-1_bal_ virus (50 ng of p24) in a total of 100 µl VEC tissue growth media for 1 h before applying to the apical surface of the EpiVaginal™ tissues in the transwell inserts. The media from the lower chamber were then collected at different time points to determine infectivity of HIV-1_bal_ virions that have crossed the vaginal epithelial tissues by incubation of the samples with TZM-bl cells as described below.

### Production of AAV serotypes expressing GFP and b12 minibody

AAV serotypes 1, 2, 5, 6, 8, and 9 expressing GFP were produced at Harvard Gene Therapy Initiative (Harvard Institute of Medicine, Boston, MA), whereas AAV serotypes 3 and 4 expressing GFP were obtained from the AAV core at the University of North Carolina, Chapel Hill. AAV-6 expressing b12 minibody was obtained commercially (Virapur LLC, San Diego, CA).

### Construction of pTR-b12scFvFc expression vector

To construct the b12 minibody expression cassette, the b12scFv sequence was PCR amplified from a DNA plasmid kindly provided by Dennis Burton (The Scripps Research Institute, La Jolla, CA) using primers to add flanking *Sfi*I and *Not*I restriction sites for insertion into the pTRUF hingestuffer vector (described below) to yield pTR-scFvFc b12. The pTRUF hingestuffer vector was created by our laboratory as follows. The IgG1 Fc sequence (AF150959) was cloned into the pTRUF20 vector at *Not*I and *Cla*I sites. The resulting pTRUF hingestuffer vector therefore could be used to insert scFv sequences at the *Sfi*I-*Not*I sites for expression and secretion of scFvFc fusion proteins (directed by the leader sequence of the human IgG VH4 gene family) when transfected in mammalian cells alone or for production of recombinant AAV expressing the scFvFc.

The pTR-b12scFvFc vector, constructed as described, readily expressed b12 minibodies proteins which are secreted from transfected 293T cells. Typical yields of secreted protein after 3 days of transfection ranged between 10–15 µg/ml (data not shown). By SDS-PAGE gel analysis, the b12 minibody monomer was shown to be approximately 55 kDa in reducing conditions and at 110 kDa under non-reducing conditions, as expected (data not shown).

### Transduction of cells, flow cytometry analysis and fluorescence microscopy

For AAV transduction, 5×10^4^ cells were incubated in 24 well plates with AAV (1, 2, 3, 4, 5, 6, 8, 9)-GFP (10^10^ genomic copies) for 4 h with the VK2/E6E7, Ectl/E6E7 and Endl/E6E7 cells or overnight for primary cells. Subsequently, the medium was replaced, and the cells were examined on day 3. Expression of GFP protein was detected by flow cytometry (FACS Calibur, Becton Dickinson) and represented as percentage of GFP positive cells, as well as visually assessed by fluorescence microscopy. To evaluate transduction of the stem cell population in huPGECs with AAV-6-GFP, the dissociated cells were immunostained for surface Ck17 (Dako, CA) and nuclear p63 (Santa Cruz Biotechnology, CA) cervical stem cell markers [26]. The GFP positive cells were sorted using a FACSAria (Becton Dickinson) instrument after gating on Ck17+p63+double positive cells, and images were captured under a fluorescence microscope.

### Immunohistochemical staining of paraffin-embedded human vaginal, ectocervical and endocervical tissues

This study was approved by the Institutional Review Boards of Boston University Medical School and Brigham and Women's Hospital Boston MA. All tissue samples were fixed in a solution of 95% ethanol and 5% acetic acid and processed for embedding in wax. For immunohistochemical staining 5micron sections were cut, mounted on glass slides then de-waxed and rehydrated followed by a blocking step with a serum –free protein solution (Dako, Carpinteria, CA,USA) for 30 min.This was drained from the sections and then they were incubated with the rabbit primary antibody p63 (Santa Cruz, CA, USA) at a dilution of 1∶50 for 60 mins.Sections were then washed in Tris-buffered saline containing 0.1% Tween 20 (TBST).The primary antibody was detected by incubating the sections with a proprietary secondary reagent (alkaline phosphatase Envision Dako) for 30 mins.followed by 2×5 min. washes in TBST. Finally the antibody was visualized by treating the sections with a substrate for alkaline phosphatase (Fast Red Dako) that stains positive cells a bright red. After washing the sections were counterstained with aqueous hematoxylin mounted in a glycerin-based medium and cover slipped.

### Isolation of huPGECs

Human cervical tissue samples were obtained from patients who had undergone hysterectomies. These tissues were procured with informed consent by the National Disease Research Interchange (NDRI, Philadelphia, PA). All tissues were placed in cold DMEM medium with gentamicin (Invitrogen), and epithelial cells were isolated within 24 h of surgical removal. The procedure for isolation of epithelial cells was based on a previously described protocol [24] with modifications. Briefly, each tissue was minced into 1- to 2-mm pieces, digested in 1 mg/mL of collagenase-dispase containing 1 mg/mL of DNase (Sigma) for 3 h at 37°C with gentle stirring. Following digestion, the mixture was passed through a 40 µm cell strainer, centrifuged (100×*g* for 20 min) and resuspended in DMEM 10% FCS. After an additional centrifugation step, the pellet was resuspended in keratinocyte serum-free medium in T-25 flasks. The huPGECs were passaged twice prior to use or frozen in aliquots to be thawed when needed.

### Production of b12 minibodies and gp120 ELISA binding assay

To produce the b12 minibodies, 293T cells were transfected with pTR-b12scFvFc using Lipofectamine 2000 (Invitrogen) according to the manufacturer's instructions. Sixteen hours after transfection, the media was replaced, and the cells were further incubated for another 48 h. The supernatant was then harvested, sterile filtered and purified after overnight incubation at 4°C with Protein A agarose beads (GE Healthcare, Piscataway, NJ) according to the manufacturer's instructions. The b12 minibodies proteins were eluted with IgG elution buffer (Thermo Scientific, Waltham, MA) and buffer exchanged in PBS using Amicon Ultra-15 centrifugal filtration units (30 kDa molecular weight cut-off; Millipore, New Bedford, MA).

The concentrations of the purified b12 minibodies and the full-length b12 IgG1 (NIH-ARRRP) were measured using a human IgG ELISA kit (Bethyl Laboratories, Montgomery, TX), and both proteins were tested at equimolar concentrations for the capacity to bind to HIV-1 bal gp120 (NIH-ARRRP) by ELISA. Ninety-six-well microtiter plates were coated overnight at 4°C with 20 ng/well of HIV-1 bal gp120 in 0.05 M carbonate-bicarbonate buffer (pH 9.6, Sigma) and then blocked in PBS (1% BSA) for 1 h. Serial dilutions of equimolar amounts of b12 IgG or b12 minibodies were added to the plate for 1 h at room temperature. After washing, HRP-conjugated, affinity-purified goat anti-human IgG (Bethyl Laboratories, Montgomery, TX) was added (1∶50,000) for 1 h. After extensive washing, the plate was developed by addition of TMB substrate (Kirkegaard & Perry Laboratories, Gaithersburg, MD) and detected by reading the absorbance (OD) at 450 nm.

### HIV-1 neutralization assay

Neutralization assays were performed by pre-incubation of serially diluted IgG/minibodies with virus (100 TCID_50_) for 1 h in 100 µl of culture media (DMEM 10% FBS) prior to addition to TZM-bl cells (approximately 50% confluent) in a final volume of 200 µl in 96-well plates. After 48 h, the cells were lysed in 50 µl passive lysis buffer (Promega, Madison, WI) per well, and luciferase activity was measured using the Luciferase Assay System (Promega) on an automated luminometer (Berthold Technologies, Bad Wildbad, Germany). The percent neutralization of a given antibody concentration was calculated by: (Relative luciferase activity in the absence of antibody – luciferase activity in the presence of a given antibody concentration/luciferase activity in the absence of antibody)×100.

### Measurement of viral infectivity

TZM-bl cells, containing a luciferase gene under the control of the HIV-1 LTR promoter, were seeded in 96-well plates (4000 cells/well) and grown overnight. The next day, the medium was removed, and the cells were incubated with 100 µl of either neat or diluted media collected at different time points up to 24 h from the lower chambers of the human vaginal organotypic VEC transwell cultures. After 48 h of incubation, the cells were then washed, lysed, and luciferase activity was measured as described above for HIV-1 neutralization assays.

## Results

### Identification of the optimal AAV serotype for transduction of immortalized human female genital epithelial cell lines

**Figure 1:**
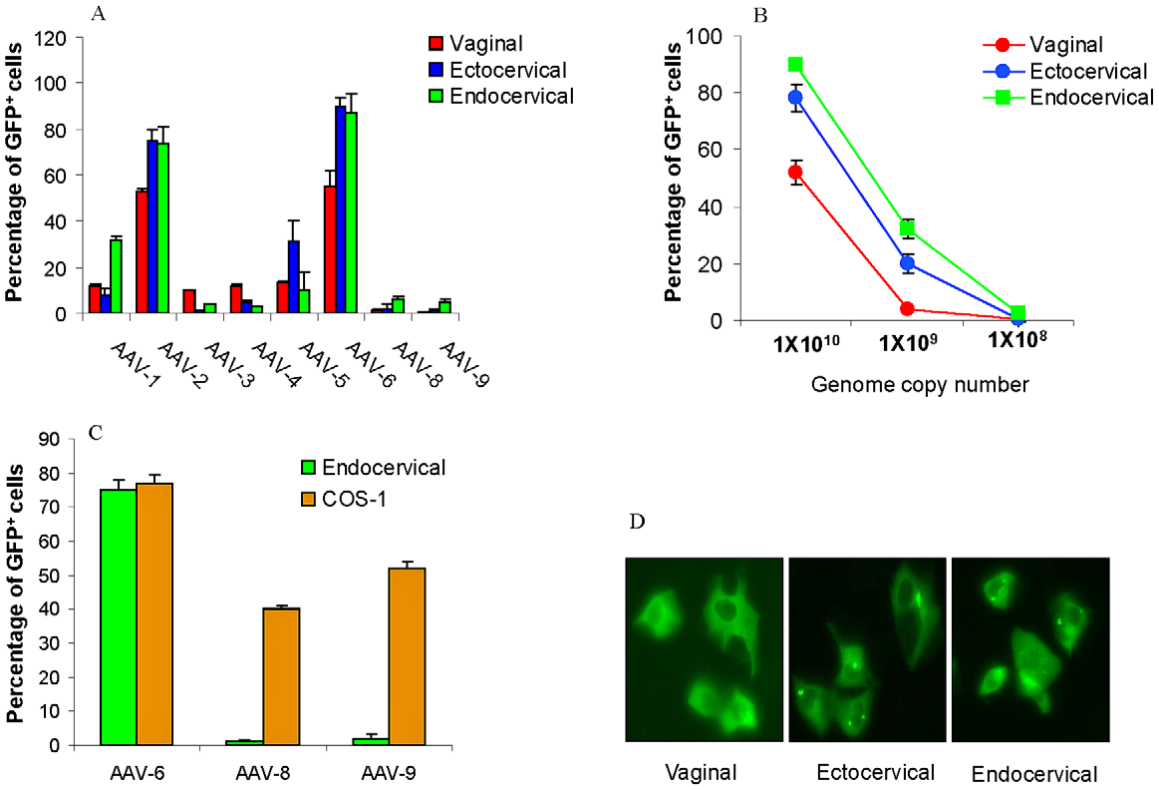
Transduction of human endocervical, ectocervical and vaginal epithelial cells by various AAV serotypes expressing GFP. (A) Expressions of GFP protein by transduced cells were detected by FACS and presented as percentages of GFP positive cells. Note that AAV-2 and AAV-6 yielded the highest transduction rates. (B) A dose dilution of AAV-6-GFP vector. (C) AAV-8-GFP and AAV-9-GFP transduction of COS-1 cells. (D) Visual assessment of AAV-6-GFP transduction by fluorescence microscopy of vaginal, ectocervical and endocervicel cell lines.

To identify the most optimal AAV serotype for transduction of female genital cells, the efficiencies of AAV 1, 2, 3, 4, 5, 6, 8 and 9 expressing GFP were evaluated by transducing immortalized human endocervical (Endl/E6E7), ectocervical (Ectl/E6E7) and vaginal (VK2/E6E7) epithelial cell lines. Relative transduction efficiencies were evaluated by flow cytometry (Fig. 1A) as well as visual assessment by fluorescence microscopy (Fig. 1D). AAV-1, AAV-2, AAV-5 and AAV-6 were found to transduce the endocervical, ectocervical and vaginal epithelial cell lines with AAV-2 and AAV6 being the most efficient. By contrast, the transduction efficiencies of AAV-3, AAV-4, AAV-8 and AAV-9 serotypes were low in these lines. In a dose dilution experiment using AAV-6-GFP vector, transduction efficiencies of the three genital epithelial cell lines were compared over a 100-fold range from 1×10^10^ to 1×10^8^ genomic copies (Fig. 1B); 1×10^10^ genomic copies gave the highest gene transfer efficiency and showed no evidence of cytotoxicity (data not shown) and was chosen as the optimal transducing units for our study. To confirm that the inability of AAV-8 and AAV-9 to transduce the female genital cell lines was due to their tropism for the specific cell type and not to a defect in the vectors themselves, COS-1 cells were tested for transduction. As shown in Fig. 1C, AAV-8-GFP and AAV-9-GFP were able to transduce COS-1 cells effectively, indicating that both vectors were functional.

### Transduction of human primary genital-epithelial cells (huPGEC) using AAV-6GFP

**Figure 2:**
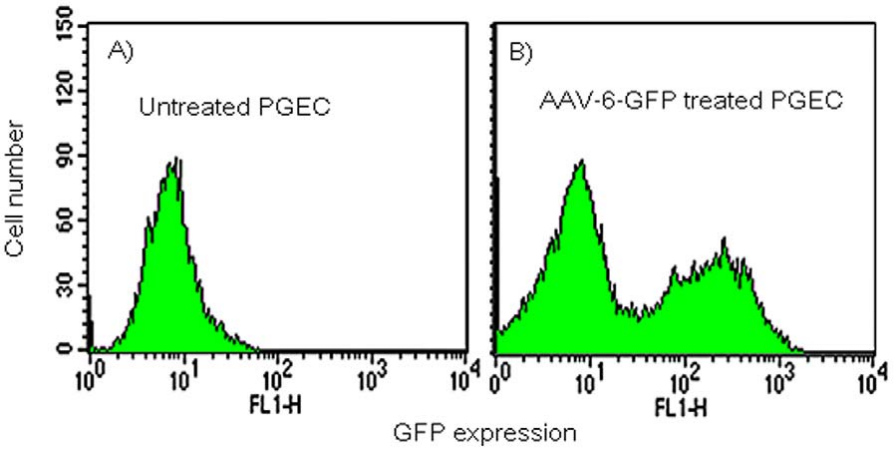
AAV-6-GFP transduction of human primary genital epithelial cells was assessed by flow cytometry to detect GFP expression. (A) untreated cells. (B) transduced with AAV-6-GFP.

Based on the experiments above (Fig. 1A, B), AAV-6-GFP was chosen as the most efficient serotype to test transduction of huPGEC. Single cell suspensions of huPGEC were prepared from discarded cervical tissues from anonymous hysterectomy patients and cultured in serum-free keratinocyte medium as described in Materials and Methods. Only low passage (2–3 passages) huPGEC were used for transduction. Cells were exposed to AAV-6-GFP and found to be efficiently transduced as determined from flow cytometric analysis of total primary cervico-vaginal cells (Fig. 2A-B) and visual assessment by fluorescence microscopy (data not shown).

### Transduction of female human primary genital epithelial stem cells with AAV-6-GFP

**Figure 3:**
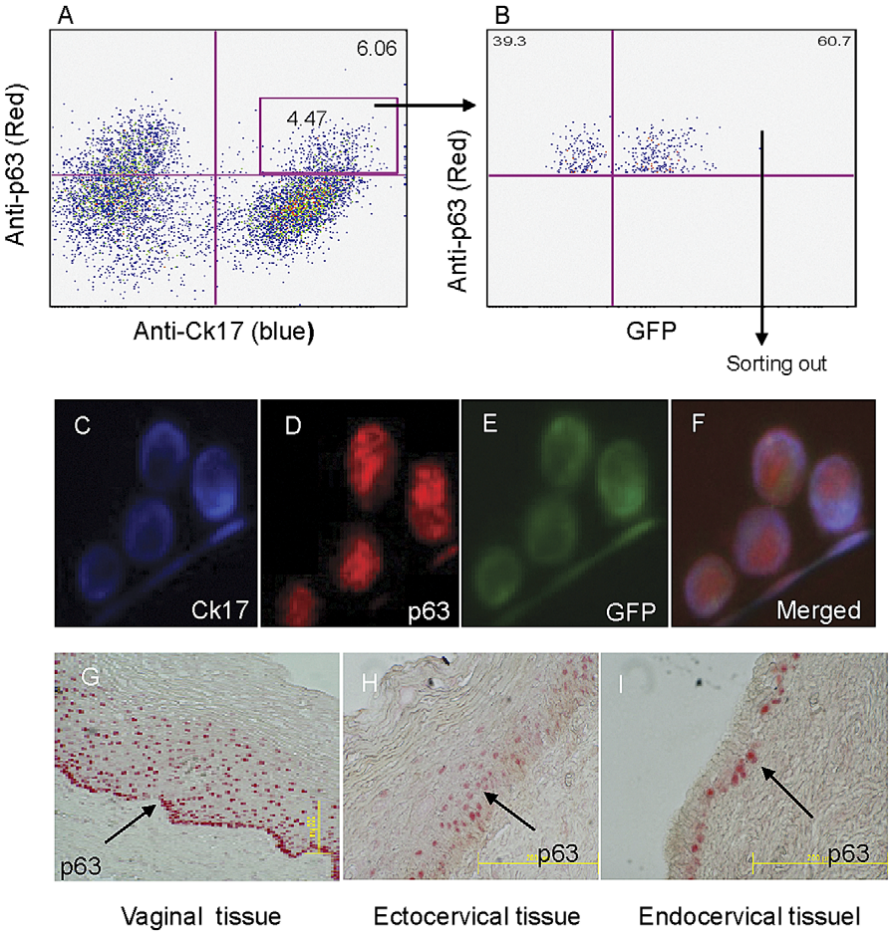
Transduction of human genital epithelial stem cells by AAV-6-GFP. At 3 days after exposure to AAV-6-GFP, cells were stained with anti-Ck17 and anti-p63 antibodies. (A) The stained cells were analyzed by flow cytometry by gating first on CK17/p63 double positive cells (A) and then on the GFP-positive cells within this population (B). The AAV-6-GFP transduced cells were then sorted for further examination by fluorescence microscopy. A representative cluster of cells displaying all three distinct colors are shown: (C) blue (anti-ck17), (D) red (anti-p63) and (E) green (GFP). (F) Merged image of C, D and E. (G-I) Immunohistochemical staining of epithelial stem cells in vaginal, ectocervical and endocervical tissues. The p63 positively stained cells are mainly located in the basal epithelial cell layer. Note that in the endocervix (I), the epithelium is composed of a single cell thick layer under which the epithelial stem cells are located.

Since terminally-differentiated apical vaginal epithelial cells continuously shed, targeting the genital epithelial stem cells for transduction by AAV would be ideal for stable and durable gene transfer *in vivo*. Accordingly, we examined whether human genital epithelial stem cells could be transduced by AAV-6-GFP *in vitro*. The isolated huPGECs were exposed to AAV-6-GFP overnight and then stained with antibodies against the epithelial stem cell markers, anti-CK17 and nuclear anti-p63 [25]. The stained cells were analyzed by flow cytometry by gating first on CK17/p63 double positive cells (4.47%) (Fig. 3A) and then on the GFP-positive cells within this population (Fig. 3B). The AAV-6-GFP transduced cells were then sorted for further examination by fluorescence microscopy (Fig. 3C-F). Representative images of cells displaying the epithelial cell marker CK17 (blue), stem cell marker p63 (red) and GFP (green) are shown in Figure 3C-E (individual signals) and Figure 3D (merged image). These data demonstrate that AAV-6-GFP can successfully transduce cells with reported epithelial stem cell characteristics.

The vaginal and ectocervical stratified epithelial layers are on average about 40 cells thick (Fig. 3G-I), and it would be challenging for the AAV vector to reach the epithelial stem cells in the basal layer. Therefore, AAV vector access to this stem cell population could be enhanced by synchronous delivery timed to the secretory phase of the menstrual cycle when thinning of the ecto/vaginal epithelium is most evident [27]. Fortunately, the endocervix is only one cell layer thick above the epithelial stem cells (Fig. 3I), which is more accessible to the AAV vector during the entire menstrual cycle.

### Expression and functional evaluation of b12 minibodies

**Figure 4:**
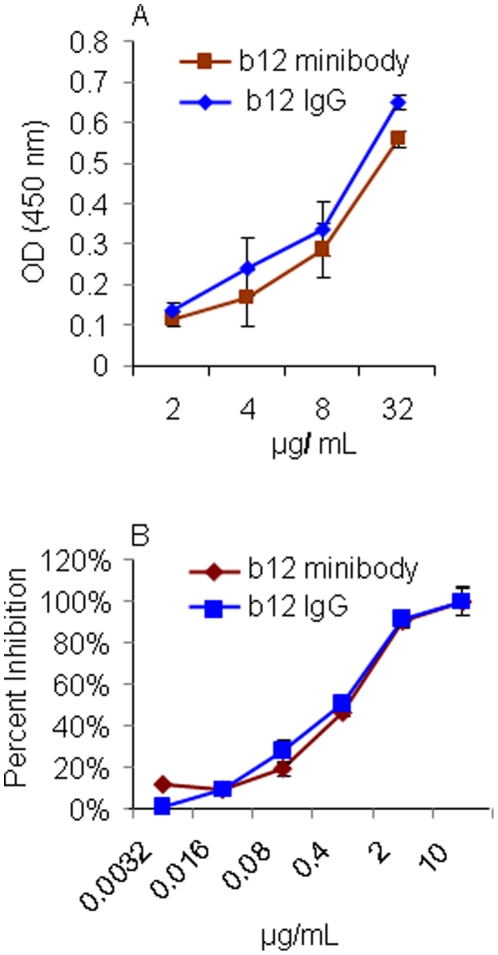
Functional comparison of b12 minibodies and full-length b12 IgG. Both b12 minibodies and full-length b12 IgG proteins were tested at equimolar concentrations for their capacity to (A) bind to HIV-1 gp120 by ELISA, and (B) to neutralize HIV-1_bal_ virus.

To evaluate the biological activity of b12 minibody in comparison to that of full-length b12 IgG1, the minibodies were produced by transient transfection of pTR-b12scFvFc into 293T cells and affinity-purified from the cell culture supernatants using Protein A. Both proteins were tested at equimolar concentrations for the capacity to bind to HIV-1_bal_ gp120. Figure 4A shows that b12 IgG and the b12 minibody have very similar ELISA binding curves at the concentrations used in this assay.

The capacity of b12 minibodies to neutralize HIV-1 infection in comparison to full-length b12 IgG1 was also evaluated. Briefly, serially diluted antibodies and 100 TCID_50_ HIV-1_bal_ were mixed in 100 µl total volumes in culture media (quadruplicate samples for each dilution) and pre-incubated for 1 h at 37°C. The Ab-virus mixtures were then incubated with TZM-BL cells containing a luciferase gene under the control of the HIV-1 LTR promoter, in 96-well plates and incubated for 2 days before measuring reporter activity of cell lysates. As shown in Figure 4B, b12 minibody displayed identical virus neutralization activity compared with the full-length b12 IgG1. For both antibodies, the calculated IC_90_ (90% Inhibitory Concentration) was 2 µg/ml, and the IC_50_ (50% Inhibitory Concentration) was 0.4 µg/ml in this assay against the HIV-1_bal_ strain. These data confirm that the b12 minibodies can neutralize HIV-1 similarly to the full-length b12 antibodies.

### b12 minibodies interfere with HIV-1 transfer and infectivity in the VEC tissue model

**Figure 5:**
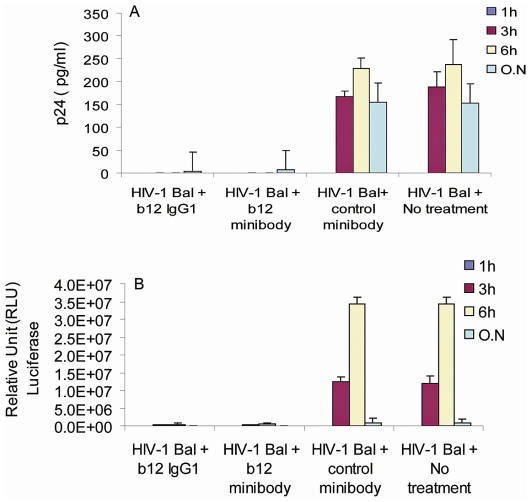
Inhibition of HIV-1 transfer and activity by b12 minibodies in the human VEC organotypic model tissues. After a 1 h pre-incubation of b12 minibodies or full-length b12 IgG (10 µg/ml) with HIV-1_bal_ (50 ng), medium from the basal chambers were collected at different time points and tested for inhibition of HIV-1_bal_ transfer by measuring p24 content by ELISA (A) and for inhibition of virus infectivity by incubation on TZM-bl target cells (B). Note that media collected at 3 and 6 h from tissue samples treated with HIV-1_bal_ and b12 IgG1 antibodies or with b12 minibodies had almost completely lost their ability to infect TZM-bl cells. Irrelevant 11 A minibodies served as negative controls.

As the b12 minibody was confirmed to have the same capacity as full-length b12 IgG1 to neutralize HIV-1 infectivity (Fig. 4B), its activity was further compared with the full-length b12 IgG1 in preventing HIV-1 transfer and subsequent infectivity. An organotypic vaginal epithelial cells transwell tissue model (VEC tissue) was used as described in Materials and Methods. After confirming the integrity of the VEC tissues using exclusion of 70 kDa Dextran-Rhodamine from the lower chamber, b12 minibodies, full-length b12 IgG1 or irrelevant control minibodies (each 10 µg/ml) were incubated with or without HIV-1_bal_ virus (50 ng) and then added to the apical surface of the organotypic VEC tissue in transwell inserts. The media from the lower chambers were then collected at different time points to measure the amounts of viral particles that have crossed the VEC tissues and to determine if they were infectious. The amounts of viral particles were measured by p24 ELISA (Fig. 5A), and infectivity was tested by incubation of the samples with the TZM-bl cells and measuring luciferase gene activity as discussed previously (Fig. 5B).

In the absence of an HIV-1-specific Ab or in the presence of an irrelevant Ab, HIV-1_bal_ was capable of penetrating the transwell system, as measured by p24 ELISA (Fig. 5A), and remained infective after crossing the VEC tissues (Figure 5B). By contrast, in the presence of either b12 minibodies or full-length b12 IgG1, HIV-1 transfer and infectivity were inhibited (Figure 5 A-B), which is in agreement with published data on the ability of b12 to block early HIV-1 transfer by blocking HIV-1 viral particle binding to syndecans expressed on the vaginal epithelial cell surface [12], [15]. Interestingly, samples collected after 24 h from the control samples had detectable amounts of p24, but failed to show infectivity in TZM-bl cells, which is likely due to the presence of antimicrobial peptides in epithelial cell secretions [3], [7] and/or shortened half-life of cell-free virus when they fail to rapidly encounter target cells [9]. These findings demonstrate that b12 minibodies are comparable to full-length b12 IgG1 in their ability to inhibit HIV-1 transfer through vaginal epithelial cells Student's *t* test was performed and the results were found statistically significant (*P*-value <0.001).

### Inhibition of HIV-1 transfer through VEC tissues and infectivity following transduction with AAV-6 expressing b12 minibody

**Figure 6:**
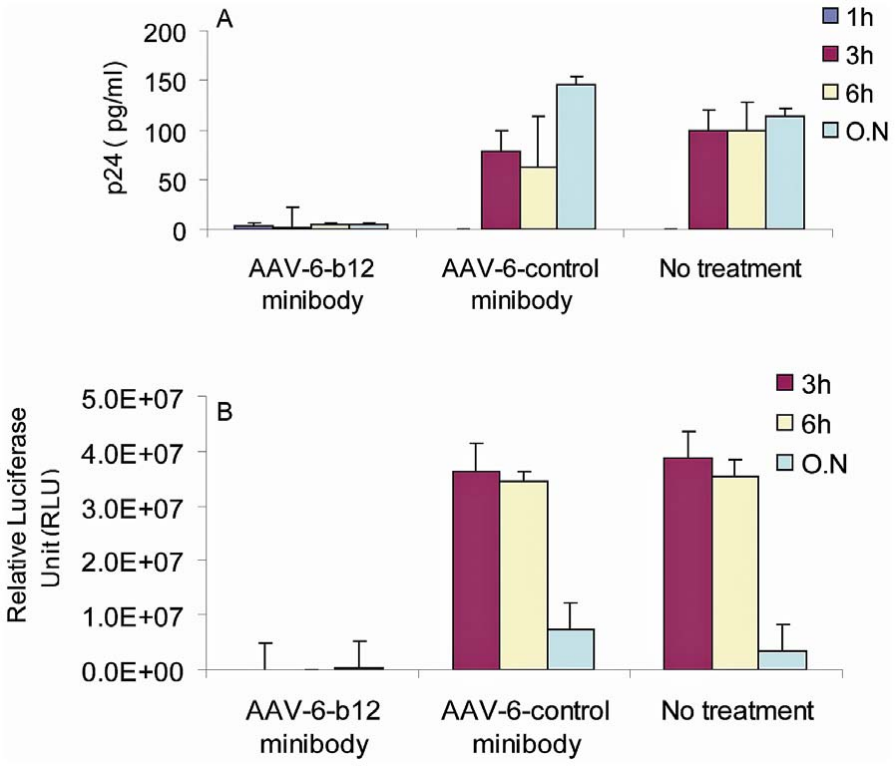
Inhibition of HIV-1 transfer and activity in the human VEC organotypic model tissues transduced with AAV-6 expressing b12 minibodies. AAV-6-b12 minibodies or AAV-6-11A minibodies (control) at 5×10^10^ particles was applied to the upper layers of the VEC tissues for 24 h for transduction. Four days after the transduction, HIV-1_bal_ (50 ng) was applied to the upper layers of the tissues, and medium from the basal chambers were collected at various timepoints and tested for inhibition of viral transfer (A) and infectivity (B) as in Fig. 5.

In assessing the AAV-6 vectors, we first wanted to determine the levels of b12 minibodies that could be secreted from AAV transduced cells in the VEC model. For these studies, we obtained commercially produced and CsCl purified AAV-6-b12minibody and control AAV-6-11A minibody The human VEC tissues in transwells were transduced by applying AAV-6 encoding b12 minibody or the negative control 11A minibody (5×10^10^ genomic copies) to the apical surface of the tissues. Twenty-four hours later, the tissues were washed and cultured for four days before use in the blocking assay. Using a quantitative IgG ELISA, we confirmed that >12 µg/ml of b12 minibody was secreted into the upper chamber of the transwell at day 5 after transduction (data not shown). Cell-free virus transfer and *in vitro* infectivity after virus challenge were examined in the AAV transduced VEC tissue model. HIV-1_bal_ virus (50 ng) was applied to the apical layer, and media from the bottom chamber was sampled at 1 h, 3 h, 6 h and overnight. The AAV-6-b12 minibody transduced tissue effectively blocked transfer of virus to the lower chamber, while the level of virus transferred in the AAV-6-negative control transduced tissues were not significantly different from that of the untreated control (no AAV transduction) (Fig. 6A). Importantly, the supernatants in the lower chamber of the tissues transduced with AAV-6- -b12 minibody contained little or no virus particles and were not infectious, while those of the tissues that were untreated or transduced with the negative control AAV had high levels of infectious virus in the lower chambers at the 3 and 6 h time points (Fig. 6B). This data demonstrates that recombinant AAV-6 can be used to deliver bNAb/minibodies to primary cervical and vaginal epithelial cells and protect against HIV-1 challenge *in vitro*.

Student's *t* test was performed and the results were found statistically significant (*P*-value <0.001).

## Discussion

This study provides evidence that AAV-mediated gene transfer of the human anti-gp120 b12 minibody to primary cervical and vaginal epithelial cells can protect against virus challenge *in vitro*. To date, there is no highly effective microbicide against HIV-1 infection. Although a recent trial showed that a tenofovir containing vaginal gel could reduce HIV-1 infection rates by 39% [28], daily application of the gel or before and after sexual acts was impractical for many users and adherence to the use of the gel dropped over time. Such behavior-related issues may be avoided by delivering anti-HIV agents with more durable activity. This present study provides proof-of-principle for a novel microbicide strategy against HIV-1 utilizing an AAV vector, that potentially offers long-term stable transduction of cervico-vaginal stem cells with secretion of a potent and broadly neutralizing anti-HIV gp120 minibody.

In preclinical animal models of human disease, AAV vectors have emerged as a favored gene transfer system due to their safety profile and the potential to transduce non-dividing cells. To date there have been 12 different serotypes of AAV isolated from human and non-human primates [29]. Different AAV serotypes are capable of transducing a wide variety of tissue types, including muscle, lung, brain and eye [17], [18], [19], [20], [21], [22]. However, specific AAV transduction of mucosal tissues in the female genital tract has not yet, to our knowledge, been published. In the present study, AAV-GFP gene transfer studies were performed on immortalized human endocervical, ectocervical and vaginal epithelial cell lines as well as huPGEC. Among the 8 serotypes tested, AAV-2 and 6 were the most efficient for transduction of these cells (Figure 1A). In this study, AAV-6 was chosen over AAV-2 serotype because AAV-6 (a hybrid of AAV-1 and AAV-2) has shown lower immunogenicity than AAV-2 [30], and so may provide significant advantages over AAV-2 for gene transfer to female genital epithelial stem cells.

The choice of utilizing the b12 mAb in this study was based on evidence from both *in vitro* and *in vivo* studies. The *in vitro* studies demonstrated that both b12 IgG1and IgA2 are able to inhibit transfer of cell-free HIV-1 to ME-180 cells and are able to block viral attachment to and uptake by epithelial cells [15]. Additional evidence from macaque models indicate that the broadly neutralizing antibody b12 IgG1 is capable of conferring protection against SHIV infection when administered by intravenous or intravaginal (topical) routes [16], [31]. In the present study b12 antibody was produced in the scFvFc (minibody) format as opposed to full-length IgG for AAV gene transfer for several reasons. First, as AAV vectors do not package more than ∼5 kb of foreign DNA efficiently, the AAV vector cannot accommodate conventional antibody expression cassettes to drive the mAb heavy chains and light chains from two individual promoters [32], [33] although, 2A sequences have been used successfully to express full-length IgG [34], [35]. Second, the minibodies are generally expressed at high levels in mammalian cells, and third, the smaller size of the minibodies may allow greater tissue penetration and targeting of neutralizing epitopes that may be inaccessible or sterically hindered as a whole IgG [36], [37].

The generated b12 minibodies were confirmed to be as potent as the full-length b12 IgG both in their capacity to bind to HIV-1 gp120 and to neutralize HIV-1_bal_ virus (Fig. 4A, B). Furthermore, b12 minibodies were able to block HIV-1 transfer and infectivity of cell-free virus transferred through VEC tissues (Figs. 5 and 6). These data are in agreement with previously published studies [15], which showed that b12 IgG inhibited the transfer of cell free HIV-1 through the human cervical epithelial cell line ME-180 and that inhibition was due to the ability of IgG b12 to block both viral attachment to and uptake by epithelial cells. In addition, studies on rhesus macaques [16], [31] demonstrated that b12 IgG applied vaginally can afford protection against SHIV challenge. Thus, the data clearly demonstrate that the activity of b12 minibodies is comparable to the full-length b12 antibodies in their capacity to inhibit *in vitro* transfer of cell free HIV-1 through human VEC tissues. The findings also strongly suggest that b12 minibodies should protect the vaginal mucosa of rhesus macaque against SHIV challenge in planned future studies.

The present study also demonstrates that primary genital epithelial stem cells could be transduced *in vitro*. This same strategy should work effectively *in vivo* if the AAV vector has ready access and sufficient time to transduce the epithelial stem cell populations. There are two distinct epithelial stem cell populations that differ in morphology and location. The endocervix is naturally thin, and there is only a one cell thick layer above the epithelial stem cells (Fig. 3I). We propose that this population of columnar epithelial stem cells may be the most straightforward and efficient to transduce *in vivo*. In contrast, the *in vivo* transduction of squamous epithelial stem cells of the vaginal and ectocervix could be less efficient due to their thick layers (Fig. 3G-I), numerous tight junctions [38] and their continuous shedding. It could be challenging for the AAV vectors to reach the ectocervical and vaginal epithelial stem cells located in the basal layer. To overcome this, AAV vectors could be applied during the secretory phase of the menstrual cycle when mucosa is dominated by progesterone and the cervico-vaginal layer is at its thinnest [25] or alternatively the vector could be applied with a gene gun [39], or on gently abraided tissue. These approaches could facilitate AAV vector penetration and transduction of epithelial stem cells in the basal layer. To achieve stable and durable gene transfer *in vivo* and to create a milieu rich in BnAbs, transduction of the genital epithelial stem cells in both endocervical and ectocervical/vaginal locations may be essential for replenishing a clinically relevant number of b12 BnAb secreting cells.

In summary, this study represents a novel HIV-1 microbicide strategy that is both feasible and attractive in light of the absence of a highly effective prophylactic HIV-1 vaccine. The results provide proof of concept that AAV-BnAb gene transfer to primary cervical and vaginal epithelial cells can protect against HIV-1 challenge *in vitro.* Importantly, demonstration of AAV mediated transduction of cervico-vaginal epithelial stem cells highlights the potential of this work as a means to convey durable and long-lasting expression of BnAbs against HIV-1 in the female genital tract. Further studies are required to examine the effectiveness of this strategy in primate models, including testing additional BnAbs and syndecan decoys such as those recently reported [7], [14] before moving this approach into human clinical trials. If clinically relevant levels of anti-HIV biological agents can be achieved *in vivo*, this approach may not only provide protection against HIV-1 infection but a means by which local neutralization escape, a hallmark of HIV-1 pathogenesis, can be inhibited.
